# Knee joint dislocations—Current epidemiology and treatment in Germany

**DOI:** 10.1002/ksa.12519

**Published:** 2024-10-26

**Authors:** Johannes Weber, Dominik Szymski, Lorenz Huber, Josina Straub, Volker Alt, Julia Elisabeth Lenz

**Affiliations:** ^1^ Department of Trauma Surgery University Medical Center Regensburg Regensburg Germany

**Keywords:** arthroplasty, dislocation, epidemiology, joint, knee

## Abstract

**Purpose:**

Knee joint dislocations, though rare, present significant challenges due to potential complications like vascular and nerve damage, and are known to be often linked to sports injuries, accidents and obesity. This study aims to analyse the epidemiology, incidence and treatment approaches for knee dislocations in Germany from 2019 to 2022.

**Methods:**

This retrospective cohort study utilized data from the German Institute for the Hospital Remuneration System to examine knee dislocation cases across German medical institutions. Patient data coded under International Statistical Classification of Diseases and Related Health Problems 10 for ‘knee dislocation’ enabled detailed analysis by age, sex and surgical procedures categorized by operation and procedure codes. The Patient Clinical Complexity Level (PCCL) assessed complication severity.

**Results:**

Analysis of 1643 knee dislocation cases revealed an incidence rate of 0.44–0.54 per 100,000 inhabitants annually. During the years 2020 and 2021, there were fewer cases of knee dislocations. Male patients comprised 50%–56% of cases, with an average hospital stay of 11 days. Most cases were PCCL 0 (62%–72%) and predominantly affected patients aged 18–29 years. Anterior tibial dislocations were common among classified cases. Injuries included ligament ruptures, meniscus lesions and grade I soft‐tissue injuries. Patients with pre‐existing knee prostheses constituted 0%–16% annually. Treatment involved closed reduction, external fixation and surgeries like capsuloligamentous reconstructions and arthroscopic procedures. Revision knee arthroplasty was required in 2%–9% of cases, with obesity rates up to 7%.

**Conclusions:**

This study provides valuable insights into the epidemiology, incidence and treatment of knee dislocations in Germany, with a focus on demographic risk factors, treatment complexities and the impact of obesity and knee prostheses. The findings emphasize the importance of specialized care in larger hospitals, comprehensive management of concomitant injuries and the need for improved coding accuracy. Future research should aim to refine treatment protocols.

**Level of Evidence:**

Level III.

**Abbreviations**
BMI: body mass index
ICD‐10: International Statistical Classification of Diseases and Related Health Problems 10
InEK: German Institute for the Hospital Remuneration System
OPS: operation and procedure codes
PCCL: patient clinical complexity level

## INTRODUCTION

A knee joint dislocation occurs when there is a complete disruption of the tibiofemoral joint, leading to the dislocation of the tibia from the femur. This rare injury is characterized by its high potential for concomitant damage to the popliteal artery and peroneal nerve, which can result in limb‐threatening ischaemia and long‐term functional impairment if not promptly addressed [8, 10, 12, 17, 18, 24, 30].

General risk factors for knee joint dislocations include sports, motor vehicle collisions, falls from significant heights and occupations involving heavy physical labour [18, 26, 33]. Furthermore, obesity is a risk factor for low‐velocity knee dislocations and a predictor of poorer outcomes [21, 29, 32].

The management of knee joint dislocations typically involves urgent reduction of the dislocation to restore joint alignment, followed by a thorough assessment of vascular and neurological status [22, 25, 31]. Treatment approaches can vary from nonoperative methods, such as external braces and physical therapy, to surgical interventions aimed at repairing or reconstructing the injured ligaments and addressing any neurovascular injuries [5, 9, 11, 15, 19]. In patients following knee arthroplasty, revision surgery with exchange of the prosthesis and implantation of a rotating hinge total‐knee prosthesis is the most common treatment [1, 28].

This study aims to provide detailed information about the epidemiology and incidence of knee joint dislocations in Germany over the years 2019–2022 and an overview of current treatment practices.

## MATERIALS AND METHODS

This retrospective cohort study analyses all cases of knee joint dislocations and the surgical treatments performed at German medical institutions between 2019 and 2022, as provided by the German Institute for the Hospital Remuneration System, ‘InEK—Institut für das Entgeltsystem im Krankenhaus’. The InEK system collects data from hospitals through routine documentation of patient treatments. These data include diagnosis codes, procedures and treatment information, which hospitals submit for reimbursement purposes. The InEK uses this information to analyse healthcare costs, evaluate resource use and adjust hospital reimbursement rates accordingly. Patient data associated with the ICD‐10 codes for ‘knee dislocation’ (S83.10, S83.11, S83.12, S83.13, S83.14 and S83.18) were used to identify hospitalized patients with a knee dislocation during this four‐year period. This allowed for a detailed epidemiological analysis focused on age groups and sex. For all cases with a primary diagnosis of knee dislocation, surgical treatment coding (OPS codes = operation and procedure codes) was used to report the type of procedure performed. The minimum number of patients per group required to be listed in the ‘InEK’ registry was five patients.

All diagnoses of knee dislocations between 2019 and 2022 were included in the study analysis.

The InEK reported the Patient Clinical Complexity Level (PCCL), which is calculated through a complex procedure based on secondary diagnosis values. It indicates the severity of complications or comorbidities with results ranging from 0 (low complexity) to 6 (high complexity) [7, 23].

Categorical data are expressed as frequency counts (percentages). Data were analysed using the statistical software SPSS Version 26.0 (IBM, SPSS Inc.). The mean age and standard deviation for 2019–2022 were calculated using weighted age midpoints and case distributions. Age group midpoints were multiplied by the percentage of cases to compute the weighted mean, and the weighted variance was used to calculate the standard deviation.

## RESULTS

Patient demographics are shown in Table 1. The total number of knee dislocation cases included in the study from 2019 to 2022 was 1643, with a mean of 410.75 cases per year. The incidence of knee dislocations in Germany during this period was between 0.44 and 0.54 per 100,000 inhabitants. Male patients made up an average of 53% of the cases. The average length of stay for patients was 10.9 days, with a mean standard deviation of 12.7 days. Patients with a knee prosthesis implanted before the injury comprised 0%–16% in the respective years 2019–2022. The overall mean age across all four years is 48.47 years, with a standard deviation of 21.22 years.

**Table 1:** Knee dislocation cases.

Year	Number of cases	Incidence per 100,000 inhabitants	Male patients (%)	Age (mean, SD, days)	Average length of stay (days)	Implanted knee prosthesis (%)
**2019**	420	0.51	56	46.39 ± 20.73	10.9 ± 14.4	11
**2020**	395	0.47	50	49.34 ± 21.75	10.8 ± 11.0	13
**2021**	367	0.44	54	50.39 ± 21.44	10.7 ± 11.4	16
**2022**	451	0.54	50	47,76 ± 20.70	11.2 ± 14.0	0

*Note*: Percentages are given as the respective patient proportion in relation to the total patients included in each year. Averages are given as means with standard deviations.

**Figure 1:**
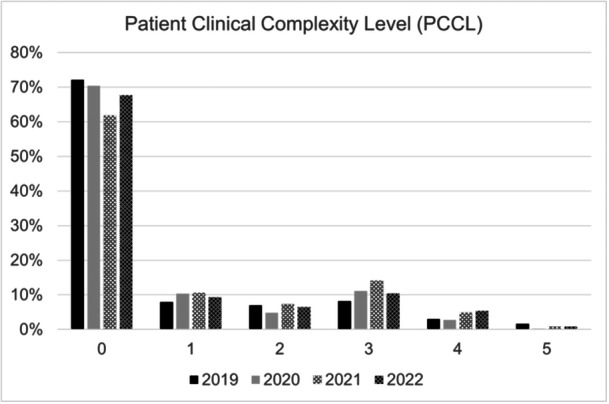
Patient clinical complexity level (PPCL). Percentages are given as the respective patient proportion in relation to the total patients included in each year.

The PCCL is shown in Figure 1. Most patients, ranging from 62% in 2021 to 72% in 2019, were categorized at PCCL 0.

**Figure 2:**
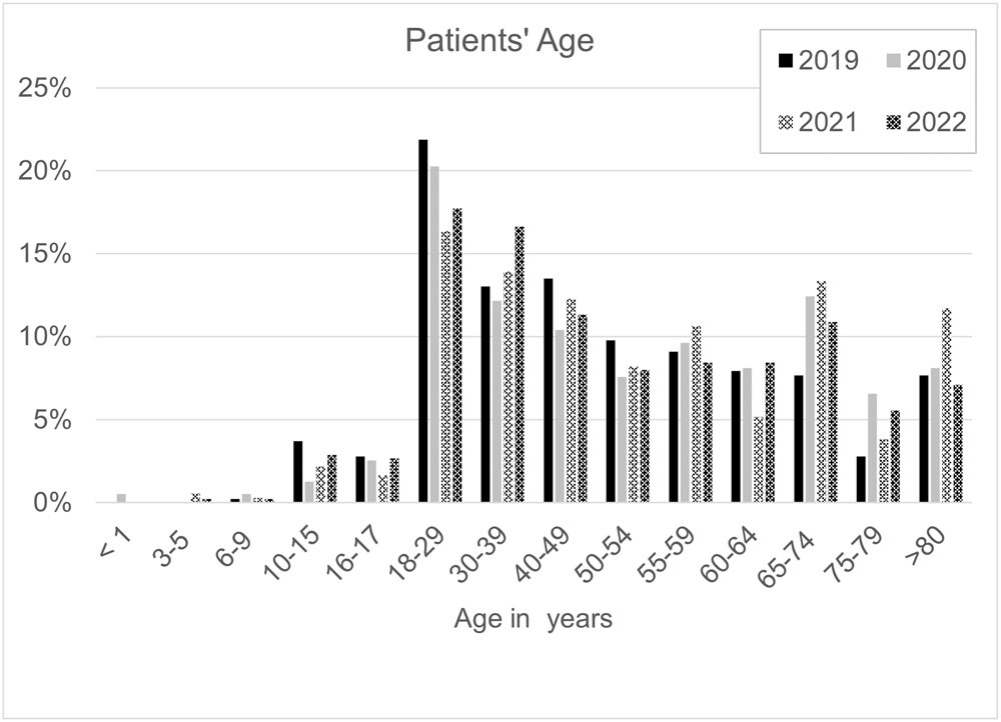
Patients' age distribution. Percentages are given as the respective patient proportion in relation to the total patients included in each year.

The patients' age distribution is shown in Figure 2, with most patients (16%–22%) falling into the 18–29 years age category. A second peak was seen in patients older than 65 years.

About 2%–4% of patients had Grade I obesity (body mass index [BMI] 30–34,9 kg/m²), 1%–9% had Grade II obesity (BMI 35–39,9 kg/m²) and 0%–7% had Grade III obesity (BMI > 40 kg/m²).

**Figure 3:**
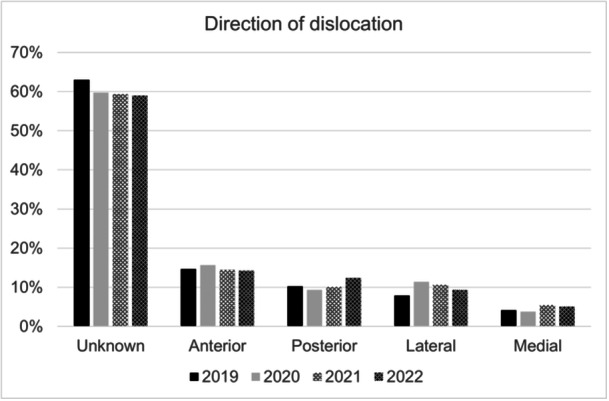
Direction of dislocation. Percentages are given as the respective patient proportion in relation to the total patients included in each year.

The direction of dislocation is shown in Figure 3. About 59%–63% of patients were not classified regarding the direction of dislocation. Among those classified, the most common type of dislocation was anterior tibial dislocation with 14%–16% of cases.

The concomitant injuries are listed in Table 2. The most common concomitant injuries were cruciate and collateral ligament ruptures (19%–44%), meniscus lesions (19%–24%) and closed soft‐tissue injuries (7%–34%). Concerning the closed soft‐tissue injuries, most injuries were classified as I° (30%–34%).

**Table 2:** Concomitant injuries.

	2019 (%)	2020 (%)	2021 (%)	2022 (%)
Ligament rupture				
Anterior cruciate ligament	39	44	42	43
Posterior cruciate ligament	37	41	37	37
Lateral collateral ligament	22	24	25	24
Medial collateral ligament	19	24	22	19
Intraarticular lesion				
Meniscus	19	19	20	24
Cartilage	1	1	4	5
Fracture				
Tibia	11	13	17	16
Fibula	4	7	6	6
Femur	5	5	6	8
Neurovascular injury				
Nervus peronaeus	7	12	10	13
Arteria poplitea	7	5	8	8
Emboly/thrombosis	4	2	4	3
Soft tissue injury				
I°, closed	31	30	32	34
II°, closed	16	20	18	17
III°, closed	9	7	7	8
I°, open	1	1	0	1
II°, open	0	0	1	2
III°, open	1	1	2	2

*Note*: Percentages are given as the respective patient proportion in relation to the total patients included in each year.

The performed procedures are shown in Table 3. Regarding the initial surgeries following the damage control principle, most patients were initially treated via closed reduction (33%–77%). About 43%–48% of patients treated via closed reduction were retained via fixateur externe. The reconstructions of the medial (26%–30%) and lateral capsuloligamentous complexes (22%–25%), as well as combined ligament reconstructions (6%–25%), were among the most common open surgeries. The most frequent arthroscopic procedure was joint irrigation with arthrolysis (11%–16%). A revision of a total knee arthroplasty was necessary in 2%–9% of cases, whereas the primary implantation of a knee prosthesis occurred in only 3% of all patients in 2020. Neurolysis was a relatively common procedure, performed in 5%–8% of patient cases.

**Table 3:** Procedures performed.

	2019 (%)	2020 (%)	2021 (%)	2022 (%)
Damage control surgery				
Closed reduction without fixateur externe	18	22	26	24
Open reduction without fixateur externe	1	2	0	1
Closed reduction with fixateur externe	15	17	21	22
Open reduction with fixateur externe	2	3	3	4
Open surgery				
Joint irrigation and synovectomy	3	7	8	8
Suture—medial capsuloligamentous complex	26	26	27	30
Suture—lateral capsuloligamentous complex	25	24	22	25
Suture—anterior cruciate ligament	8	6	9	9
Suture—posterior cruciate ligament	10	9	10	10
Bony refixation—anterior cruciate ligament	3	3	3	5
Bony refixation—posterior cruciate ligament	4	6	4	5
Reconstruction—anterior cruciate ligament	2	0	0	0
Reconstruction—posterior cruciate ligament	2	0	2	0
Reconstruction—medial patellofemoral ligament	1	3	2	1
Combined ligament reconstruction	25	6	8	9
Meniscus refixation	5	5	4	5
Arthroscopic surgery				
Joint irrigation and arthrolysis	11	15	16	16
Augmentation—anterior cruciate ligament	0	0	2	0
Augmentation—posterior cruciate ligament	3	5	3	4
Reconstruction—anterior cruciate ligament	7	7	5	8
Reconstruction—posterior cruciate ligament	7	5	4	7
Chondroplasty	2	2	2	7
Meniscus refixation	7	6	4	9
Partial meniscectomy	4	4	4	5
Arthroplasty‐related operations				
Revision total knee arthroplasty	2	7	9	5
Total knee arthroplasty implantation	0	3	0	0
Neurovascular operations				
Neurolysis	8	8	5	8
Thrombectomy—popliteal artery	1	1	2	1
Venous interposition graft—popliteal artery	1	0	4	4
Other operations				
Wound debridement	13	4	7	12
Secondary suture	2	3	3	6
Tendon suture	5	11	5	7
Fasciotomy	4	3	3	4
Vacuum assisted closure therapy	9	3	5	9
Tibial plate osteosynthesis	5	3	2	5

*Note*: Percentages are given as the respective patient proportion in relation to the total patients included in each year.

**Figure 4:**
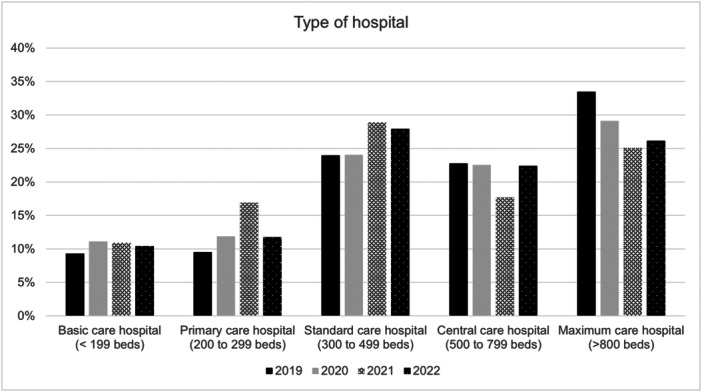
Type of hospital. Percentages are given as the respective patient proportion in relation to the total patients included in each year.

The types of hospitals where patients were treated are shown in Figure 4. Patients were treated in larger hospitals with more than 300 beds.

## DISCUSSION

The study provides critical insights into knee dislocations in Germany from 2019 to 2022. The incidence of knee dislocations ranged from 0.44 to 0.54 per 100,000 inhabitants. Concomitant injuries were common, with cruciate and collateral ligament ruptures affecting 19%–44% of patients, while meniscus lesions were present in 19%–24% of cases. Closed soft‐tissue injuries, particularly I° injuries, were observed in up to 34% of patients. Anterior tibial dislocation was the most frequently classified direction (14%–16%). The majority of patients (33%–77%) underwent closed reduction as part of damage control surgery, with 43%–48% requiring a fixateur externe.

The incidence of knee dislocations in Germany, ranging from 0.44 to 0.54 per 100,000 inhabitants, reflects the relative rarity of this injury. During the COVID‐19 lockdown periods in Germany in 2020 and 2021, there were fewer knee dislocations. This is likely due to the accident mechanism, which is characterized by workplace accidents and high‐risk sports that did not take place during the lockdowns. The consistency in incidence rates before and after the COVID‐19 lockdowns suggests a stable pattern of occurrence. In the existing literature, incidences are rarely described due to the rarity of the pathology [13]. The near‐equal distribution of male and female patients indicates that knee dislocations affect both genders almost equally [3].

The age distribution data reveal a bimodal pattern, with most patients falling into the 18–29 years age category and a second peak in patients over 65 years old. The younger cohort is likely affected by high‐energy trauma from sports or accidents, while older adults likely experience dislocations related to pre‐existing knee prostheses or falls [4]. This highlights the need for clinicians to apply age‐specific preventative measures. Younger individuals may benefit from sports safety programs, while older adults, particularly those with knee prostheses, require close monitoring for fall risks. The findings regarding the predominance of anterior tibial dislocations align with existing literature, where hyperextension is often cited as the main mechanism of injury [3, 27].

The average length of hospital stay for patients remained relatively stable, fluctuating slightly around 11 days. This consistency suggests that treatment protocols and recovery times have not significantly changed, despite potential variations in patient demographics or hospital practices. The PCCL data show that the majority of patients were categorized at PCCL 0, indicating a lower level of clinical complexity for most cases. This could reflect effective initial management and stabilization of knee dislocations, minimizing complications.

Concomitant injuries, including cruciate and collateral ligament ruptures and meniscal lesions, reflect the complexity of knee dislocations, requiring a comprehensive diagnostic and therapeutic approach. The high incidence of ligament ruptures (19%–44%) emphasizes the importance of using advanced imaging techniques, such as MRI, to properly assess injury severity [18]. Multiligament reconstruction should be considered in severe cases to restore knee stability. These findings are consistent with previous studies that highlight the complexity of treating knee dislocations with multiple ligamentous injuries [16, 20]. The presence of meniscus injuries in up to 24% of patients further underscores the need for a meticulous surgical approach.

Neurovascular injuries, particularly affecting the peroneal nerve and popliteal artery, were observed in a significant proportion of cases, emphasizing the critical importance of early neurovascular assessment. These injuries are consistent with reports from other studies and require timely intervention to prevent long‐term complications such as permanent nerve damage or limb‐threatening ischaemia [4, 5, 17]. Clinicians should maintain a high level of suspicion for these injuries in knee dislocation patients to ensure appropriate and timely management.

Surgical management trends in this study indicate that most patients (33%–77%) initially underwent closed reduction, which is the standard of care for stabilizing dislocations while minimizing further soft tissue damage. However, a significant proportion of these cases required additional stabilization with external fixation, suggesting that knee dislocations are often complex injuries requiring staged management [15]. The frequent performance of open surgeries for ligamentous repair, including medial and lateral capsuloligamentous reconstructions, supports the need for comprehensive surgical planning to address multiple injured structures. This aligns with best practices in treating knee dislocations, which often require multiligament reconstruction to restore function and stability [2, 9, 10, 13, 19].

The relatively high rate of joint irrigation with arthrolysis (11%–16%) indicates a preference for minimally invasive surgical techniques, which may reduce recovery times and minimize postoperative complications. The requirement for revision total knee arthroplasties (TKA) in 2%–9% of cases points to the challenges of managing dislocations in patients with pre‐existing knee prostheses [1, 14, 28]. Clinicians should be aware of the increased complexity of such cases and consider early referral to specialized centres for these patients. The need for neurolysis in 5%–8% of cases highlights the importance of addressing nerve injuries, which are a common and serious complication of knee dislocations. Prompt neurolysis is essential to optimize patient outcomes, as these nerve injuries can have long‐term functional consequences [4, 17].

Most patients were treated in larger hospitals with more than 300 beds, suggesting that severe knee dislocations are typically managed in well‐equipped facilities capable of providing specialized care. This finding suggests that clinicians in smaller centres should consider referring complex cases to higher‐volume hospitals for optimal care. Additionally, the study notes a relatively high prevalence of obesity among patients, particularly in those with Grade II and III obesity. This underscores the need for clinicians to consider obesity as a significant factor in both the incidence and management of knee dislocations. Obesity not only increases the risk of injury but also complicates both surgical management and rehabilitation, suggesting that weight management strategies should be integrated into long‐term patient care [6].

Our study has several limitations. One major drawback of all registry studies is that the analysis relies on the coding of diseases (ICD‐10) and procedures (OPS). Errors in coding, such as misclassification, could not be identified. However, the data provided contain extensive information about all patients treated for knee dislocation in German hospitals within the specified time frame. Another limitation is that treatment details could not be closely correlated with patient data, such as comorbidities or ASA scores, preventing a thorough risk and outcome analysis. Furthermore, it should be noted that patients treated on an outpatient basis (particularly those with Schenk I and potentially Schenk II/III) were not included in the analysis. Therefore, errors may occur since only inpatient data were available.

## CONCLUSION

This study provides valuable insights into the epidemiology, incidence and treatment of knee dislocations in Germany, with a focus on demographic risk factors, treatment complexities and the impact of obesity and knee prostheses. The findings emphasize the importance of specialized care in larger hospitals, comprehensive management of concomitant injuries and the need for improved coding accuracy. Future research should aim to refine treatment protocols.

## AUTHOR CONTRIBUTIONS

*Conceptualization*: Julia Elisabeth Lenz and Johannes Weber. *Methodology*: Julia Elisabeth Lenz. *Formal analysis and investigation*: Julia Elisabeth Lenz. *Writing—original draft preparation*: Julia Elisabeth Lenz and Johannes Weber. *Writing—review and editing*: Dominik Szymski, Lorenz Huber, Josina Straub and Volker Alt. *Supervision*: Johannes Weber and Volker Alt.

## CONFLICT OF INTEREST STATEMENT

The authors declare no conflict of interest.

## ETHICS STATEMENT

The authors have nothing to report.

## Data Availability

Data and statistical analysis are available from the authors upon reasonable request.
